# Maternal Pre-Pregnancy Body Mass Index, Gestational Weight Gain and Children’s Cognitive Development: A Birth Cohort Study

**DOI:** 10.3390/nu14214613

**Published:** 2022-11-02

**Authors:** Xuemei Hao, Jingru Lu, Shuangqin Yan, Fangbiao Tao, Kun Huang

**Affiliations:** 1 Department of Maternal, Child and Adolescent Health, School of Public Health, Anhui Medical University, Hefei 230032, China; 2 Key Laboratory of Population Health Across Life Cycle, Ministry of Education of the People’s Republic of China, Anhui Medical University, Hefei 230032, China; 3 NHC Key Laboratory of Study on Abnormal Gametes and Reproductive Tract, Hefei 230032, China; 4 Anhui Provincial Key Laboratory of Population Health and Aristogenics, Hefei 230032, China; 5 Ma’anshan Maternal and Child Health Center, Ma’anshan 243011, China; 6 Scientific Research Center in Preventive Medicine, School of Public Health, Anhui Medical University, Hefei 230032, China

**Keywords:** pre-pregnancy, body mass index, gestational weight gain, children, cognitive development, public health

## Abstract

To investigate the joint effect of maternal pre-pregnancy body mass index (BMI) and gestational weight gain (GWG) on children’s cognitive development. We recruited 1685 mother–child pairs from the Ma’anshan Birth Cohort in China. Pre-pregnancy BMI and GWG were calculated based on the height and weights measured at multiple antenatal checkups. Children’s cognition was assessed by Wechsler Preschool and Primary Scale of Intelligence-Fourth Edition. Poisson regression model was used to analyze the association between maternal pre-pregnancy BMI and children’s cognitive dimensions under different GWG categories. Women with overweight or obese before pregnancy were more likely to obtain excessive GWG. When women had excessive GWG, pre-pregnancy overweight was associated with low children’s PSI (*OR* = 1.69, *95%CI*: 1.02–2.81) and pre-pregnancy obesity was related to poor VCI in children (*OR* = 3.71, *95%CI*: 1.49–9.22), after adjusting for potential confounders. In pre-pregnancy underweight mothers, adequate GWG reduced the risk of below-average VSI in children (*OR* = 0.22, *95%CI*: 0.05–0.92), but excessive GWG was related to low FSIQ in children (*OR* = 2.53, *95%CI*: 1.34–4.76). In women with excessive GWG, maternal pre-pregnancy BMI displays an inverted U-shape association with children’s cognition. Moreover, adequate GWG in women with pre-pregnancy underweight was beneficial for children’s cognition.

## 1. Introduction

Gestational weight gain (GWG) is usually defined as the change in weight measured before pregnancy or during the first trimester of pregnancy to the end of pregnancy (before childbirth) [1]. In 2009, the Institute of Medicine (IOM) issued revised guidelines for healthy GWG based on maternal pre-pregnancy BMI [2]. In recent years, studies have found that the incidence of excessive GWG is as high as 50% in reproductive-age women and in pregnant women [3,4], and about one-quarter (25.6%) of the population is obese before pregnancy [5]. Maternal obesity before pregnancy or excessive GWG during pregnancy can lead to a variety of adverse pregnancy outcomes, such as hypertensive events, gestational diabetes, emergency cesarean section and macrosomia [1,6,7].

Weight gain during pregnancy may relate to pre-pregnancy BMI, but it is not well defined. There was a study that suggested overweight or obese women had lower weight gain compared to normal weight women [8]. However, researchers also argued that overweight and obese women were more likely to exceed recommended weight gain than normal-weight women [3]. Anyway, it has been well established that maternal pre-pregnancy overweight and obesity or excessive GWG affects offspring’s neurodevelopmental outcomes, particularly in the area of cognitive development [9,10,11,12]. A cross-sectional study of 778 Chinese children aged 7–14 years indicated that maternal obesity before pregnancy was strongly associated with children’s lower cognition and sociality [13]. The Avon Longitudinal Study of Parents and Children (ALSPAC) found that maternal pre-pregnancy obesity was negatively correlated with children’s intelligence quotient [14]. The results of the Millennium Cohort study also showed a negative association between maternal pre-pregnancy BMI and cognitive ability in children aged five and seven years and revealed a stronger relationship with children’s increasing age [15]. The Maternal Health Practices and Child Development cohort study observed that children whose mothers had higher GWG took longer to complete executive functional tasks at 10 years old [16], and higher maternal GWG was associated with lower academic achievement scores in reading and spelling [17].

However, there is great controversy on the association between maternal GWG and children’s cognitive development. The Columbia Center for Children’s Environmental Health Mothers and Newborns Study has used Wechsler Intelligence Scale for Children (WISC-IV) to assess the cognitive levels of seven-year-old children. It revealed that, in boys, maternal overweight and obesity were related to low full-scale intelligence quotient (FSIQ) and perceptual reasoning scores, and maternal overweight was associated with low processing speed scores, but maternal GWG was not associated with cognitive development among boys [18]. A longitudinal cohort study in Norway and Sweden also found that maternal GWG has nothing to do with children’s cognition [19]. Furthermore, we found that most studies just examined the relationship between isolated pre-pregnancy BMI or isolated GWG exposure and children’s cognitive development.

Therefore, based on a large-sample birth cohort, we aimed to investigate the joint effect of maternal pre-pregnancy BMI and GWG on offspring’s cognitive development. We hope to raise public health implications for women’s health in reproductive age to maintain a healthy BMI before pregnancy and appropriate weight gain during pregnancy.

## 2. Materials and Methods

### 2.1. Study Population

The current study was based on the Ma’anshan Birth Cohort (MABC), a large sample prospective study designed to examine the association between early life exposure and maternal and children’s health. Pregnant women who underwent their first antenatal checkup at the Ma’anshan Maternal and Child Health Care Center from May 2013 to September 2014 were recruited. The inclusion criteria were as follows: (1) maternal age ≥18 years old; (2) within 14 gestational weeks; (3) planned to have antenatal checkups and childbirth at Ma’anshan MCH Care Center; (4) being able to understand and complete the questionnaire; (5) being willing to be followed up. A total of 3474 pregnant women who met the criteria were recruited in the cohort.

**Figure 1:**
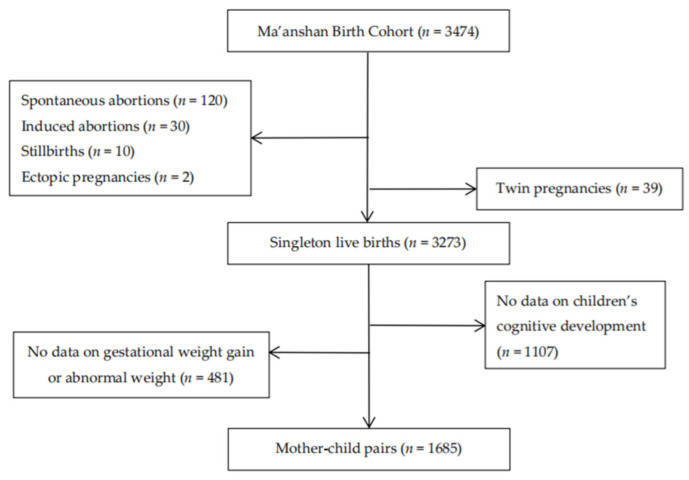
Flow chart of recruited participants.

In data analysis, 201 participants were excluded due to spontaneous abortion, induced abortion, stillbirth, ectopic pregnancy and twin pregnancy. A total of 3273 singleton live births were included in the study. After further excluding women without GWG data or with abnormal weight and children who did not take cognitive development tests, a total of 1685 mother–child pairs were included in final data analysis (Figure 1). The basic characteristics of the mother–child pairs included in the analysis and excluded from the analysis are shown in Table S1.

This study was approved by the Ethics Committee of Anhui Medical University (Number: 20131401). All participants understood the purpose of the study and signed informed consent.

### 2.2. Assessment of Maternal Pre-Pregnancy BMI and GWG

Women’s height and weight were measured at their first antenatal checkup, and BMI was calculated according to the formula BMI (kg/m^2^) = weight (kg)/[height (m)]^2^ and regarded as pre-pregnancy BMI. We categorized the pre-pregnancy BMI into four groups according to the World Health Organization classification: underweight (BMI < 18.5 kg/m^2^), normal weight (18.5 kg/m^2^ ≤ BMI < 25.0 kg/m^2^), overweight (25.0 kg/m^2^ ≤ BMI < 30.0 kg/m^2^) and obesity (BMI ≥ 30.0 kg/m^2^) [20].

Maternal weight was measured before childbirth, and then the GWG was calculated by subtracting the weight measured at the first antenatal checkup from the weight before childbirth. The revised GWG guidelines published by IOM in 2009 pointed out that women with underweight before pregnancy should gain 12.5–18.0 kg during pregnancy, women with normal weight should gain 11.5–16.0 kg, women with overweight should gain 7–11.5 kg and obese women should gain 5–9 kg [2]. According to the recommended range of weight gain by IOM guidelines for different pre-pregnancy weights, we classified maternal GWG into inadequate GWG, adequate GWG and excessive GWG based on women’s pre-pregnancy BMI categories.

### 2.3. Children’s Cognitive Development Assessment

At a mean age (SD) of 55.6 (6.9) months old, children’s cognitive development was assessed by using the Chinese version of Wechsler Preschool and Primary Scale of Intelligence-Fourth Edition (WPPSI-Ⅳ CN), which has high reliability and validity [21,22] and can be used in children aged 2.5–6 years. In detail, children aged 30–47 months (*n* = 202) were required to complete 7 subtests to synthesize 4 indexes as verbal comprehension index (VCI, including information, receptive vocabulary and picture naming), visual space index (VSI, including block design and object assembly), working memory index (WMI, including picture memory and zoo locations) and full-scale intelligence quotient (FSIQ, calculated based on the above 3 indexes). Children aged 48–83 months (*n* = 1483) were required to complete 13 subtests to synthesize 6 indexes as VCI (three subtests were the same as 30–47 months, another was similarities), VSI (the same as 30–47 months), fluid reasoning index (FRI, including matrix reasoning and picture concepts), WMI (the same as 30–47 months), processing speed index (PSI, including bug search, cancellation and animal coding) and FSIQ (calculated based on the above 5 indexes). These indexes were based on the intelligence norm of Chinese children, which already takes into account the information of children’s age; thus, these indexes are comparable between children of different ages. We divided the calculated five subscale indexes and full-scale indexes into two categories, with 90 as the cut-off point [16]. Points less than 90 were defined as below average level, and not less than 90 was defined as average or above average level.

The cognitive test was conducted at Ma’anshan Maternal and Child Health Care Center by two investigators. They were trained by a licensed clinical psychologist who had over 10 years of clinical experience and obtained the Chinese version of the WPPSI-IV test qualification certificate. Before testing, the investigators recorded the information of child’s name, sex and age, and they were unaware of the children’s other characteristics.

### 2.4. Covariates

We used a directed acyclic graph [23] to identify the potential confounding factors in the impact of maternal pre-pregnancy BMI on the cognitive development of offspring, including maternal age at enrollment, maternal and paternal education level, maternal IQ, household monthly income per capita, maternal occupation, parity, previous adverse pregnancy outcomes, maternal smoking and maternal drinking (Figure S1).

Data on maternal age at enrollment, maternal and paternal education level, household monthly income per capita, maternal occupation, parity, previous adverse pregnancy outcomes, maternal smoking and drinking were collected by questionnaire at the first antenatal checkup. Previous adverse pregnancy outcomes included abortion, preterm birth, fetal death, stillbirth, ectopic pregnancies and previous delivery of infants with birth defects. Women who had one or more conditions were defined to have previous adverse pregnancy outcomes. Information on pregnancy complications (including pregnancy-related hypertensive disorders and gestational diabetes), gestational age at delivery, children’s birth weight and sex were extracted from the medical notes. Chinese version of Wechsler Adult Intelligence Scale (WAIS-IV) was used to assess maternal IQ. The categories of potential covariates are listed in Table 1.

Exclusive breastfeeding within 6 months after birth and the main caregivers before 3 years of age were collected as precision variables. Exclusive breastfeeding was defined according to WHO as an infant receiving only breast milk, no other liquids or solids, not even water, except for oral rehydration solution, drops or syrups consisting of vitamins, minerals supplements or medicines [24]. Main caregivers referred to the caregivers who spend more than 50% of the time and energy caring for and accompanying the children during their growth. The information was collected through children’s follow-up questionnaires at 6 and 30 months.

### 2.5. Statistical Analysis

Data entry was performed using EpiData 3.0 with a double-entry method, and a consistency check was conducted to ensure accuracy. All statistical analyses were performed by using SPSS 22.0 (Chicago, IL, USA), and a two-tailed value of *p* < 0.05 was considered statistically significant.

The demographic characteristics of the study participants were presented as mean [standard deviation (SD)] and n (%). Differences in maternal and children’s characteristics under different BMI classifications were assessed using one-way analysis of variance (ANOVA) for continuous variables and Pearson *χ*^2^ tests for categorical variables. Poisson regression model was used to analyze the association between maternal pre-pregnancy BMI and children’s cognitive dimensions under different GWG categories.

We performed four sensitivity analyses. (1) Pre-pregnancy BMI closely related to pregnancy complications [7], as might be associated with offspring’s cognitive development [25,26]. Pregnancy complications may act as a mediator between pre-pregnancy BMI and children’s cognitive function and thus were further adjusted. (2) As gestational age at delivery and birth weight were positively associated with child intelligence scores [27], we further adjusted the birth weight Z scores by gestational age. (3) As the effect of maternal pre-pregnancy BMI on children’s cognitive development may be sex-specific [18], we further adjusted the children’s sex. (4) As proved by the literature, breastfeeding has a positive effect on offspring’s cognitive development, especially in children with low cognitive test scores [28]. Meanwhile, children raised by grandparents might have poor academic performance and were more likely to have below-normal cognitive development [29]. The two variables could be the precision variables relevant to children’s cognitive function. Exclusive breastfeeding within the first 6 months after birth and the main caregivers before 3 years were therefore further adjusted.

## 3. Results

### 3.1. Demographic Characteristics of the Participants

Table 1 shows the baseline characteristics of 1685 mother–child pairs included in the study. Compared with women with normal pre-pregnancy BMI, women with pre-pregnancy overweight or obese were older (*p* < 0.001) and had a higher proportion of multiparous (*p* < 0.001). They also had higher rates of previous adverse pregnancy outcomes and pregnancy complications (*p* = 0.001 and *p* < 0.001, respectively), and their husbands were also less educated (*p* < 0.001). Children born of pre-pregnancy overweight or obese mothers had smaller gestational ages and higher birth weights (both *p* < 0.001).

### 3.2. Association between Pre-Pregnancy BMI and GWG

Table 2 shows the association between pre-pregnancy BMI and GWG in the study population. Of the mothers, 9.4% and 1.7% belonged to the category of overweight and obesity according to pre-pregnancy BMI classification, respectively. While 58.5% of pregnant women had excessive GWG during pregnancy in the total population. We found that mothers who were overweight or obese before pregnancy were more likely to gain excessive weight during pregnancy (84.3% and 82.8%, respectively) compared to pre-pregnancy underweight or normal-weight women.

### 3.3. Distribution of Children’s Cognitive Ability under Different Pre-Pregnancy BMI and GWG Categories

Table 3 shows the distribution of children’s cognitive ability under different maternal pre-pregnancy BMI in different GWG classifications. In women with inadequate GWG, a high percentage of children born of pre-pregnancy underweight mothers had below-average VSI and PSI (22.2% and 17.4%, respectively). In women with adequate GWG, children born of pre-pregnancy underweight mothers had the lowest rate of below-average VSI (1.7%). In women with excessive GWG, approximately 12% of children born of pre-pregnancy underweight mothers were below average in several cognitive domains, respectively. A high percentage of children born of pre-pregnancy overweight mothers had below-average VSI and PSI (13.4% and 18.3%, respectively). Moreover, among children born of pre-pregnancy obese mothers, the rate of below-average VCI was highest (25.0%), followed by FSIQ (16.7%).

### 3.4. The Effect of Maternal Pre-Pregnancy BMI on Children’s Cognitive Development under Different GWG Classifications

We combined the subjects whose mothers were overweight or obese before pregnancy for analysis due to the limited sample size in the inadequate and adequate GWG group. Table 4 shows the association between different maternal pre-pregnancy BMI and children’s cognitive dimensions under different GWG classifications, the cognitive level of average or above average being the reference group. After adjusting for potential confounding factors, it showed that, in women with excessive GWG, a high risk of below-average VCI was observed in children born of pre-pregnancy obese mothers (*OR* = 3.71, *95%CI*: 1.49–9.22, *p* = 0.005). Similarly, in women with excessive GWG, children born of pre-pregnancy overweight mothers had a high risk of below-average PSI (*OR* = 1.69, *95%CI*: 1.02–2.81, *p* = 0.044).

Interestingly, in women with adequate GWG, children born of pre-pregnancy underweight mothers had a low risk of below-average VSI (*OR* = 0.22, *95%CI*: 0.05–0.92, *p* = 0.038). However, in women with excessive GWG, children born of pre-pregnancy underweight mothers had a high risk of below-average FSIQ (*OR* = 2.53, *95%CI*: 1.34–4.76, *p* = 0.004).

The sensitivity analyses did not change the main findings fundamentally (Table S2).

## 4. Discussion

In the current study, we found that excessive weight gain during pregnancy increases the risk of children’s low cognitive development. When women had excessive GWG, maternal overweight before pregnancy was associated with low children’s PSI, and maternal obesity before pregnancy was associated with low VCI in children. In pre-pregnancy underweight mothers, adequate GWG reduced the risk of below-average VSI in children, but excessive GWG was related to low FSIQ in children.

In the study population, women with overweight or obese before pregnancy were more likely to gain excessive weight during pregnancy, and the adverse effects of maternal pre-pregnancy weight on children’s cognitive development were observed in those with excessive GWG. Previous studies had similar findings. Among the 160 women with term pregnancies in the Puerto Rico Test site for Exploring Contamination Threats study, being overweight or obese at the start of pregnancy was significantly associated with excessive GWG [30]. A study based on the 2015 Pelotas Birth Cohort found that for each kilogram-unit increase in total GWG, there was a reduction of 0.007 percentiles in children’s cognitive and global neurodevelopmental scores, and children whose mothers had excessive GWG were more likely to have a developmental delay in cognitive domains [31].

Moreover, in our study, in women with excessive GWG, children born of pre-pregnancy underweight, overweight and obese mothers had a high risk of below-average cognitive development, so we suggest that maternal pre-pregnancy BMI showed an inverted U-shaped relationship with children’s cognitive development when pregnant women had excessive GWG. The findings were supported by the Collaborative Perinatal Project cohort study that revealed an inverted U-shaped relationship between maternal pre-pregnancy BMI and children’s IQ. Children had the highest IQ scores when maternal pre-pregnancy BMI was around 20 kg/m^2^ [32]. It also found that maternal obesity before pregnancy was related to low IQ in children, particularly in those whose mothers gained more than 40 lbs during pregnancy [32]. A Sweden national population-based cohort study found that only overweight and obese mothers with excessive GWG (>25 kg) were associated with impaired intelligence development in their offspring [33]. We also found that appropriate GWG in women with pre-pregnancy underweight was beneficial for children’s cognitive development, while excessive GWG was harmful. It is crucial to maintain a healthy pre-pregnancy weight and reasonable weight gain during pregnancy.

The mechanism of maternal overweight and obesity before pregnancy or excessive GWG affecting offspring’s cognitive development is not clear. A study of 1361 mother–child pairs in Project Viva found that maternal inflammation may partly mediate the association between maternal obesity and offspring’s cognitive ability [34]. The Prediction and Prevention of Preeclampsia and Intrauterine Growth Restriction study indicated that persistently high levels of maternal inflammation during pregnancy increased the risk of neurodevelopmental delay in children in cognitive, motor and social areas, and children of mothers with the highest levels of inflammation had the largest number of neurodevelopmental delay areas [35]. Maternal prepregnancy obesity or excessive weight gain during pregnancy would lead to chronic systemic inflammation and placental inflammatory response, causing increased concentrations of pro-inflammatory cytokines such as IL-6, IL-8, tumor necrosis factor-α (TNF-α) and C-reactive protein (CRP) [36,37]. Exposure to an inflammatory environment in early life affects the development and function of fetal microglia, which are related to essential neurodevelopmental functions such as neuronal proliferation and differentiation, synapse formation, myelin formation and establishment of connections [38]. Inappropriate microglia activation and changes in inflammatory levels in the hippocampus and hypothalamus may be the basis of offspring cognitive impairment caused by maternal obesity.

The effect of maternal pre-pregnancy overweight and obesity or excessive GWG on offspring’s cognitive function may also be related to dysregulated metabolic hormones. Cordner et al. demonstrated that the cognitive ability of adult male offspring of high-fat-fed dams was observed to be impaired, accompanied by reduced expression of insulin receptor and leptin receptor mRNA at weaning, as well as in adulthood [39]. Insulin receptor and leptin receptors are highly expressed in the hypothalamus, hippocampus and other brain structures, which play important roles in neurodevelopment, and are closely related to neurogenesis, synaptic morphology and synaptic plasticity, and neural circuit formation [38,40].

Studies have found that maternal inadequate GWG also affects children’s cognitive development. Priscilla et al. [33] indicated that regardless of maternal BMI in early pregnancy, the risk of intelligence development disability in children of mothers with inadequate GWG increased by about two-fold. The ALSPAC study also found that when the GWG of mothers was lower than the recommended weight, their offspring had lower entrance assessment scores and final grades [41]. However, our study did not find a relationship between maternal inadequate GWG and children’s cognitive development, which may be related to the small sample size of pregnant women with inadequate GWG in the study population.

This study has several strengths. First of all, most of the previous studies have only discussed the relationship between isolated pre-pregnancy BMI or GWG exposure and children’s cognitive development. When the exposure of the study was one of the two variables, the other was included as a potential confounder or even not included in the analysis. Based on a large-sample prospective birth cohort, we examined the effects of different pre-pregnancy BMI on children’s cognitive development under different GWG classifications, which could clarify the joint effect of maternal pre-pregnancy irrational weight and GWG on children’s cognitive development. Secondly, our study was prospectively designed; all of the variables included in the analysis were prospectively collected during continuous follow-up, including exposure, outcome and potential confounders. Meanwhile, we also considered the precision variables that could affect children’s cognitive development in the sensitivity analyses to verify the robustness and reliability of the results. In addition, the fact that the original data we used to calculate the maternal pre-pregnancy BMI and GWG were all obtained from actual measurements was also one of our strengths. In contrast, other studies had mostly used self-reported or model-predicted data [16,17,41], which might be biased from reality.

Of course, several limitations in our study have to be noted. On the one hand, the sample size of women with inadequate GWG was limited, representing only 8.2% of the total study population. The number of women with pre-pregnancy overweight or obese was also relatively low, especially for those with pre-pregnancy obesity. These conditions resulted in no sample distribution for multiple cognitive dimensions in the inadequate GWG group. Thus the estimates of the association between maternal pre-pregnancy overweight or obesity and children’s cognitive development risk were not stable in the inadequate GWG group, as was the association between maternal pre-pregnancy obesity and children’s cognitive development risk in the adequate GWG group. On the other hand, in addition to the potential confounders we included in the analysis, there were other factors could also influence pre-pregnancy BMI and children’s cognitive development, which might interfere with our findings. For example, maternal dietary patterns [42,43] and sleep quality [44,45] have been found to have an impact on the exposures and outcomes we studied. Furthermore, low paternal IQ might also be associated with an increased risk of intellectual disability in offspring [46], but the relevant data were not collected in this study.

## 5. Conclusions

The joint effect of pre-pregnancy BMI and GWG was observed on children’s cognitive development. In women with excessive GWG, maternal pre-pregnancy BMI might display an inverted U-shape association with children’s cognition. Further studies are needed to verify these findings.

## Figures and Tables

**Table 1:** Baseline characteristics of the participants.

Characteristics	Pre-Pregnancy BMI (kg/m^2^)				*p* Values
	<18.5	18.5–24.9	25.0–29.9	≥30	
**Demographic characteristics**					
Maternal educational level [*n*(%)]					0.08
Junior high school or below	40(15.3)	245(19.8)	40(25.2)	6(20.7)	
Senior middle school	72(27.6)	257(20.8)	37(23.3)	6(20.7)	
Junior college or above	149(57.1)	734(59.4)	82(51.6)	17(58.6)	
Paternal educational level [*n*(%)]					<0.001
Junior high school or below	28(10.7)	160(12.9)	41(25.8)	7(24.1)	
Senior middle school	84(32.2)	355(28.7)	43(27.0)	8(27.6)	
Junior college or above	149(57.1)	721(58.3)	75(47.2)	14(48.3)	
Household monthly income per capita (yuan) [*n*(%)]					0.46
≤2500	78(29.9)	340(27.5)	52(32.7)	11(37.9)	
2500~4000	94(36.0)	511(41.3)	62(39.0)	11(37.9)	
>4000	89(34.1)	385(31.1)	45(28.3)	7(24.1)	
**Maternal characteristics**					
Age (years) [Mean(SD)]	25.6(2.9)	26.6(3.4)	28.3(4.4)	28.0(3.8)	<0.001
IQ [Mean(SD)]	96.7(10.4)	96.3(10.8)	95.0(10.7)	99.6(9.0)	0.13
Occupation [*n*(%)]					0.50
No job	116(44.4)	485(39.2)	69(43.4)	14(48.3)	
Mental job	121(46.4)	621(50.2)	71(44.7)	11(37.9)	
Manual job	24(9.2)	130(10.5)	19(11.9)	4(13.8)	
Parity [*n*(%)]					<0.001
Nulliparous	249(95.4)	1120(90.6)	130(81.8)	22(75.9)	
Multiparous	12(4.6)	116(9.4)	29(18.2)	7(24.1)	
Previous adverse pregnancy outcomes [*n*(%)]					0.001
Did not have	150(57.5)	769(62.2)	73(45.9)	15(51.7)	
Had	111(42.5)	467(37.8)	86(54.1)	14(48.3)	
Pregnancy complications [*n*(%)]					<0.001
Did not have	245(93.9)	1044(84.5)	94(59.1)	16(55.2)	
Had	16(6.1)	192(15.5)	65(40.9)	13(44.8)	
Smoking during pregnancy [*n*(%)]					0.20
No	251(96.2)	1188(96.1)	148(93.1)	29(100.0)	
Yes	10(3.8)	48(3.9)	11(6.9)	0	
Drinking during pregnancy [*n*(%)]					0.81
No	243(93.1)	1149(93.0)	150(94.3)	28(96.6)	
Yes	18(6.9)	87(7.0)	9(5.7)	1(3.4)	
**Children’s characteristics**					
Gestational age (week) [Mean(SD)]	39.1(1.3)	39.2(1.3)	38.9(1.5)	38.0(2.7)	<0.001
Birth weight (g)^a^ [Mean(SD)]	3248.3(444.9)	3385.8(410.5)	3502.7(495.2)	3280.0(682.7)	<0.001
Children’s sex [*n*(%)]					0.97
Boy	137(52.5)	653(52.8)	84(52.8)	14(48.3)	
Girl	124(47.5)	583(47.2)	75(47.2)	15(51.7)	
Exclusive breastfeeding for 6 months ^a^ [*n*(%)]					0.98
No	228(87.4)	1093(88.4)	144(90.6)	25(86.2)	
Yes	25(9.6)	112(9.1)	14(8.8)	3(10.3)	
Main caregivers before 3 years of age ^a^ [*n*(%)]					0.85
Parents	126(48.3)	634(51.3)	80(50.3)	14(48.3)	
Grandparents	133(51.0)	594(48.1)	79(49.7)	14(48.3)	

^a^ Missing data: 1 in birth weight, 41 in exclusive breastfeeding for 6 months, 11 in main caregivers before 3 years of age. Abbreviations: BMI, body mass index; SD, standard deviation.

**Table 2:** Association between pre-pregnancy BMI and GWG [*n*(%)].

GWG	Pre-Pregnancy BMI (kg/m^2^)				*p* Value
	<18.5	18.5–24.9	25.0–29.9	≥30	
Inadequate GWG	27(10.3)	104(8.4)	4(2.5)	3(10.3)	<0.001
Adequate GWG	119(45.6)	419(33.9)	21(13.2)	2(6.9)	
Excessive GWG	115(44.1)	713(57.7)	134(84.3)	24(82.8)	

Abbreviations: GWG: gestational weight gain; BMI, body mass index.

**Table 3:** Distribution of children’s cognitive ability under different maternal pre-pregnancy BMI in different GWG classifications [*n*(%)].

GWG Classifications		Inadequate GWG				Adequate GWG				Excessive GWG			
Pre-Pregnancy BMI		<18.5	18.5~24.9	25.0~29.9	≥30	<18.5	18.5~24.9	25.0~29.9	≥30	<18.5	18.5~24.9	25.0~29.9	≥30
VCI	1	26(96.3)	95(91.3)	4(100.0)	2(66.7)	114(95.8)	394(94.0)	18(85.7)	2(100.0)	102(88.7)	671(94.1)	124(92.5)	18(75.0)
	2	1(3.7)	9(8.7)	0	1(33.3)	5(4.2)	25(6.0)	3(14.3)	0	13(11.3)	42(5.9)	10(7.5)	6(25.0)
VSI	1	21(77.8)	96(92.3)	3(75.0)	3(100.0)	117(98.3)	386(92.1)	20(95.2)	1(50.0)	99(86.1)	644(90.3)	116(86.6)	21(87.5)
	2	6(22.2)	8(7.7)	1(25.0)	0	2(1.7)	33(7.9)	1(4.8)	1(50.0)	16(13.9)	69(9.7)	18(13.4)	3(12.5)
FRI	1	23(100.0)	84(87.5)	3(75.0)	3(100.0)	98(91.6)	334(90.0)	15(93.8)	1(50.0)	90(89.1)	572(92.4)	110(91.7)	20(95.2)
	2	0	12(12.5)	1(25.0)	0	9(8.4)	37(10.0)	1(6.3)	1(50.0)	11(10.9)	47(7.6)	10(8.3)	1(4.8)
WMI	1	25(92.6)	95(91.3)	4(100.0)	2(66.7)	110(92.4)	379(90.5)	20(95.2)	2(100.0)	100(87.0)	658(92.3)	119(88.8)	22(91.7)
	2	2(7.4)	9(8.7)	0	1(33.3)	9(7.6)	40(9.5)	1(4.8)	0	15(13.0)	55(7.7)	15(11.2)	2(8.3)
PSI	1	19(82.6)	85(88.5)	4(100.0)	3(100.0)	96(89.7)	328(88.4)	14(87.5)	2(100.0)	88(87.1)	554(89.5)	98(81.7)	18(85.7)
	2	4(17.4)	11(11.5)	0	0	11(10.3)	43(11.6)	2(12.5)	0	13(12.9)	65(10.5)	22(18.3)	3(14.3)
FSIQ	1	25(92.6)	98(94.2)	4(100.0)	2(66.7)	115(96.6)	395(94.3)	19(90.5)	2(100.0)	101(87.8)	680(95.4)	123(91.8)	20(83.3)
	2	2(7.4)	6(5.8)	0	1(33.3)	4(3.4)	24(5.7)	2(9.5)	0	14(12.2)	33(4.6)	11(8.2)	4(16.7)

“1” refers to average or above average level, “2” refers to below average level. Abbreviations: GWG: gestational weight gain; BMI: body mass index; VCI: verbal comprehension index; VSI: visual space index; FRI: fluid reasoning index; WMI: working memory index; PSI: processing speed index; FSIQ: full-scale intelligence quotient.

**Table 4:** Poisson’s regression analysis of the association between different maternal pre-pregnancy BMI and dimensions of children’s cognition under different GWG classifications [*OR* (*95%CI*)].

GWG Classifications		Inadequate GWG			Adequate GWG			Excessive GWG			
Pre-Pregnancy BMI		<18.5	18.5~24.9	≥25.0	<18.5	18.5~24.9	≥25.0	<18.5	18.5~24.9	25.0~29.9	≥30
VCI	Model 1	0.43 (0.05–3.38)	Ref	1.65 (0.21–13.03)	0.70 (0.27–1.84)	Ref	2.19 (0.66–7.24)	1.92 (1.03–3.58)	Ref	1.27 (0.64–2.53)	4.24 (1.80–9.98)
	Model 2	0.59 (0.05–6.99)	Ref	1.56 (0.13–19.42)	0.80 (0.30–2.15)	Ref	2.12 (0.60–7.54)	1.78 (0.94–3.35)	Ref	1.05 (0.51–2.18)	3.71 (1.49–9.22)
VSI	Model 1	2.89 (1.00–8.33)	Ref	1.86 (0.23–14.85)	0.21 (0.05–0.89)	Ref	1.10 (0.27–4.60)	1.44 (0.84–2.48)	Ref	1.39 (0.83–2.33)	1.29 (0.41–4.10)
	Model 2	2.35 (0.67–8.29)	Ref	2.34 (0.19–28.96)	0.22 (0.05–0.92)	Ref	1.42 (0.32–6.26)	1.27 (0.73–2.20)	Ref	1.18 (0.69–2.02)	1.08 (0.33–3.51)
FRI	Model 1	-	Ref	1.14 (0.15–8.79)	0.84 (0.41–1.75)	Ref	1.11 (0.27–4.62)	1.43 (0.74–2.77)	Ref	1.10 (0.56–2.17)	0.63 (0.09–4.55)
	Model 2	-	Ref	1.32 (0.13–13.59)	0.86 (0.41–1.82)	Ref	1.14 (0.26–5.06)	1.34 (0.69–2.64)	Ref	0.87 (0.42–1.82)	0.37 (0.05–2.71)
WMI	Model 1	0.86 (0.19–3.96)	Ref	1.65 (0.21–13.03)	0.79 (0.38–1.63)	Ref	0.46 (0.06–3.31)	1.69 (0.96–2.99)	Ref	1.45 (0.82–2.57)	1.08 (0.26–4.43)
	Model 2	1.77 (0.29–10.72)	Ref	1.44 (0.13–16.00)	0.83 (0.39–1.76)	Ref	0.51 (0.07–3.81)	1.64 (0.92–2.92)	Ref	1.49 (0.82–2.70)	1.03 (0.25–4.30)
PSI	Model 1	1.52 (0.48–4.77)	Ref	-	0.89 (0.46–1.72)	Ref	0.96 (0.23–3.96)	1.23 (0.68–2.22)	Ref	1.75 (1.08–2.83)	1.36 (0.43–4.33)
	Model 2	1.95 (0.55–6.97)	Ref	-	0.83 (0.42–1.62)	Ref	1.55 (0.35–6.78)	1.19 (0.65–2.18)	Ref	1.69 (1.02–2.81)	1.19 (0.37–3.85)
FSIQ	Model 1	1.28 (0.26–6.36)	Ref	2.48 (0.30–20.57)	0.59 (0.20–1.69)	Ref	1.52 (0.36–6.42)	2.63 (1.41–4.92)	Ref	1.77 (0.90–3.51)	3.60 (1.28–10.16)
	Model 2	1.23 (0.18–8.30)	Ref	1.57 (0.11–21.85)	0.60 (0.21–1.76)	Ref	2.30 (0.52–10.22)	2.53 (1.34–4.76)	Ref	1.40 (0.68–2.89)	2.81 (0.95–8.25)

The cognitive level of average or above average was used as the reference group. “-”: no results due to the limited cases. “Ref”: reference. Model 1: Un-adjusted models; Model 2: Adjusted for maternal age at enrollment, maternal and paternal education level, maternal IQ, household monthly income per capita, maternal occupation, parity, previous adverse pregnancy outcomes, maternal smoking and drinking. Abbreviations: GWG, gestational weight gain; BMI, body mass index; VCI, verbal comprehension index; VSI, visual space index; FRI, fluid reasoning index; WMI, working memory index; PSI, processing speed index; FSIQ, full-scale intelligence quotient.

## Data Availability

The data used and/or analyzed in this study are available from the corresponding author upon reasonable request. The data are not publicly available due to restrictions of participants’ privacy.

## Supplementary Materials

The following supporting information can be downloaded at: https://www.mdpi.com/article/10.3390/nu14214613/s1, Figure S1: Directed acyclic graph to show the association between maternal pre-pregnancy BMI and children’s cognitive development; Table S1: Baseline characteristics of mother–child pairs included and excluded (*n* = 3273); Table S2: Sensitivity analyses of the association between different maternal pre-pregnancy BMI and dimensions of children’s cognition under different GWG classifications [*OR* (*95%CI*)].
