# Supplementary material for: Maternal Pre-Pregnancy Body Mass Index, Gestational Weight Gain and Children’s Cognitive Development: A Birth Cohort Study
Source: Nutrients. 2022 Nov 2;14(21):4613. doi: 10.3390/nu14214613 (PMC9654549; doi:10.3390/nu14214613)
Supplement: Supplementary file 1 [file nutrients-14-04613-s001.zip › nutrients-1943339-supplementary.pdf]

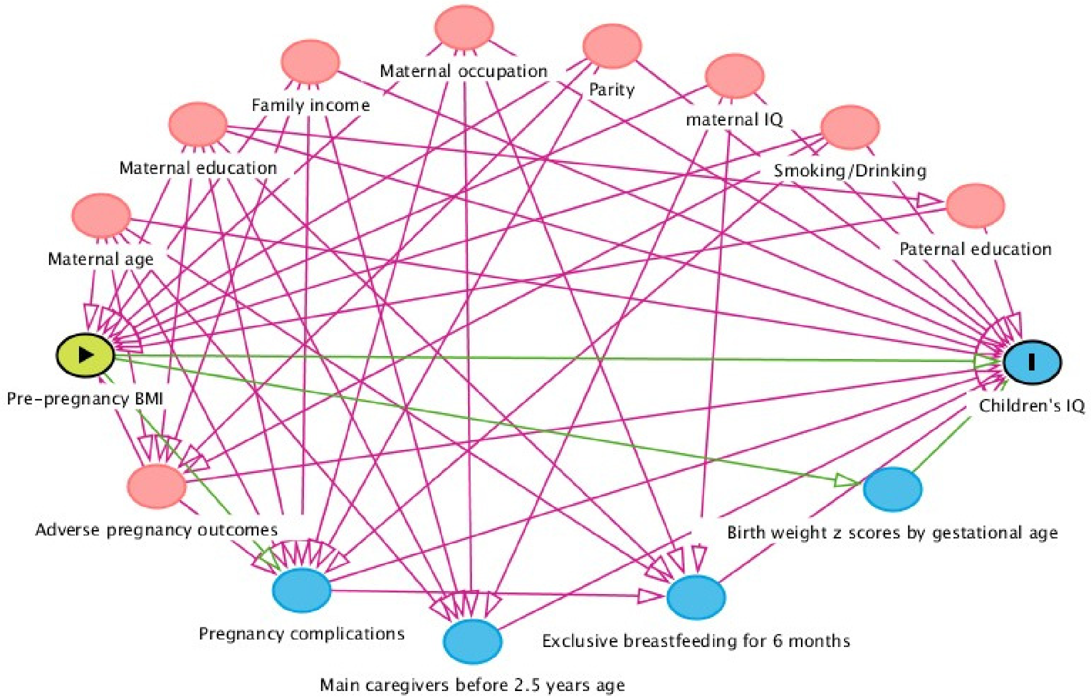


Figure S1.Directed acyclic graph to show the association between maternal pre-pregnancy BMI and children's cognitive development

**Table S1 Baseline characteristics of mother-child pairs included and excluded (*n* = 3273)**

| Characteristics | Participants included in the analysis(*n* = 1685) | Participants excluded from the analysis(*n* = 1588) | *P* values |
|---|---|---|---|
| **Demographic characteristics** | | | |
| Maternal educational level [*n*(%)] | | | 0.55 |
| Junior high school or below | 331(19.6) | 329(20.7) | |
| Senior middle school | 372(22.1) | 363(22.9) | |
| Junior college or above | 982(58.3) | 896(56.4) | |
| Paternal educational level [*n*(%)] | | | 0.17 |
| Junior high school or below | 236(14.0) | 248(15.6) | |
| Senior middle school | 490(29.1) | 421(26.5) | |
| Junior college or above | 959(56.9) | 919(57.9) | |
| Household monthly income per capita (yuan) [*n*(%)] | | | 0.003 |
| ≤2500 | 481(28.5) | 386(24.3) | |
| 2500~4000 | 678(40.2) | 724(45.6) | |
| >4000 | 526(31.2) | 478(30.1) | |
| **Maternal characteristics** | | | |
| Age (years) [Mean(SD)] | 26.6(3.5) | 26.6(3.8) | 0.85 |
| IQ [Mean(SD)] | 96.3(10.7) | 95.4(10.7) | 0.02 |
| Occupation [*n*(%)] | | | 0.15 |
| No job | 684(40.6) | 682(42.9) | |
| Mental job | 824(48.9) | 767(48.3) | |
| Manual job | 177(10.5) | 139(8.8) | |
| Parity [*n*(%)] | | | 0.66 |
| Nulliparous | 1521(90.3) | 1426(89.8) | |
| Multiparous | 164(9.7) | 162(10.2) | |
| Previous adverse pregnancy outcomes [*n*(%)] | | | 0.15 |
| Didn't have | 1007(59.8) | 910(57.3) | |
| Had | 678(40.2) | 678(42.7) | |
| Pregnancy complications [*n*(%)] | | | 0.52 |
| Didn't have | 1399(83.0) | 1305(82.2) | |
| Had | 286(17.0) | 283(17.8) | |
| Smoking during pregnancy [*n*(%)] | | | 0.48 |
| No | 1616(95.9) | 1515(95.4) | |
| Yes | 69(4.1) | 73(4.6) | |
| Drinking during pregnancy [*n*(%)] | | | 0.01 |
| No | 1570(93.2) | 1442(90.8) | |
| Yes | 115(6.8) | 146(9.2) | |
| **Children's characteristics** | | | |
| Gestational age[a] (week) [Mean(SD)] | 39.1(1.4) | 39.0(1.4) | 0.004 |
| Birth weight (g)[a] [Mean(SD)] | 3373.7(435.0) | 3356.6(460.3) | 0.28 |
| Children's sex [a] [*n*(%)] | | | 0.06 |
| Boy | 888(52.7) | 782(49.2) | |
| Girl | 797(47.3) | 801(50.4) | |
| Exclusive breastfeeding for 6 months [a] [*n*(%)] | | | 0.07 |
| No | 1490(88.4) | 1366(84.1) | |
| Yes | 154(9.1) | 171(10.8) | |
| Main caregivers before 3 years of age [a] [*n*(%)] | | | 0.02 |
| Parents | 854(50.7) | 861(54.2) | |
| Grandparents | 820(48.7) | 705(44.4) | |

a Missing data: 3 in gestational age, 8 in birth weight, 5 in children's sex, 122 in exclusive breastfeeding for 6 months, 33 in main caregivers before 3 years of age.

**Table S2 Sensitivity analyses of the association between different maternal pre-pregnancy BMI and dimensions of children's cognition under different GWG classifications [*OR* (*95%CI*)]**

| GWG classifications | | Inadequate GWG | | | Adequate GWG | | | Excessive GWG | | | |
|---|---|---|---|---|---|---|---|---|---|---|---|
| Pre-pregnancy BMI | | <18.5 | 18.5~24.9 | ≥25 | <18.5 | 18.5~24.9 | ≥25 | <18.5 | 18.5~24.9 | 25~29.9 | ≥30 |
| VCI | Model 3 | 0.81 (0.07-9.89) | Ref | 1.40 (0.12-16.80) | 0.80 (0.30-2.14) | Ref | 2.16 (0.58-8.04) | 1.76 (0.93-3.31) | Ref | 1.16 (0.55-2.44) | 4.05 (1.60-10.26) |
| | Model 4 | 0.70 (0.05-9.36) | Ref | 1.53 (0.12-20.23) | 0.82 (0.30-2.22) | Ref | 2.02 (0.56-7.31) | 1.77 (0.94-3.33) | Ref | 1.07 (0.51-2.21) | 3.74 (1.50-9.29) |
| | Model 5 | 0.55 (0.04-6.80) | Ref | 1.60 (0.12-20.75) | 0.81 (0.30-2.16) | Ref | 2.17 (0.61-7.73) | 1.79 (0.95-3.38) | Ref | 1.06 (0.51-2.20) | 3.85 (1.55-9.54) |
| | Model 6 | 0.81 (0.05-12.51) | Ref | 2.08 (0.15-28.23) | 0.82 (0.31-2.21) | Ref | 2.14 (0.57-7.99) | 1.69 (0.88-3.27) | Ref | 1.20 (0.58-2.48) | 4.21 (1.68-10.56) |
| VSI | Model 3 | 2.29 (0.63-8.24) | Ref | 2.77 (0.21-36.47) | 0.21 (0.05-0.88) | Ref | 1.79 (0.39-8.28) | 1.27 (0.73-2.20) | Ref | 1.17 (0.67-2.04) | 1.08 (0.33-3.51) |
| | Model 4 | 2.39 (0.63-9.02) | Ref | 2.34 (0.19-29.23) | 0.21 (0.05-0.89) | Ref | 1.46 (0.33-6.42) | 1.24 (0.71-2.16) | Ref | 1.21 (0.70-2.07) | 1.12 (0.34-3.63) |
| | Model 5 | 2.15 (0.59-7.83) | Ref | 2.05 (0.16-27.19) | 0.22 (0.05-0.92) | Ref | 1.60 (0.36-7.07) | 1.26 (0.73-2.18) | Ref | 1.17 (0.68-2.01) | 1.10 (0.34-3.56) |
| | Model 6 | 2.30 (0.61-8.60) | Ref | 2.51 (0.18-34.23) | 0.21 (0.05-0.90) | Ref | 1.30 (0.29-5.83) | 1.28 (0.74-2.22) | Ref | 1.15 (0.67-1.99) | 1.07 (0.33-3.50) |
| FRI | Model 3 | - | Ref | 1.29 (0.12-13.67) | 0.90 (0.43-1.92) | Ref | 0.97 (0.21-4.45) | 1.33 (0.68-2.60) | Ref | 0.97 (0.46-2.07) | 0.38 (0.05-2.86) |
| | Model 4 | - | Ref | 1.30 (0.13-12.98) | 0.78 (0.37-1.66) | Ref | 1.18 (0.27-5.21) | 1.35 (0.69-2.64) | Ref | 0.87 (0.42-1.82) | 0.37 (0.05-2.71) |
| | Model 5 | - | Ref | 1.07 (0.09-12.27) | 0.89 (0.42-1.88) | Ref | 1.59 (0.35-7.15) | 1.35 (0.69-2.65) | Ref | 0.88 (0.42-1.84) | 0.36 (0.05-2.67) |
| | Model 6 | - | Ref | 1.47 (0.14-16.06) | 0.89 (0.42-1.89) | Ref | 1.27 (0.28-5.74) | 1.27 (0.63-2.56) | Ref | 0.88 (0.42-1.85) | 0.37 (0.05-2.75) |
| WMI | Model 3 | 1.69 (0.27-10.46) | Ref | 1.30 (0.12-14.81) | 0.82 (0.39-1.74) | Ref | 0.55 (0.07-4.22) | 1.62 (0.91-2.87) | Ref | 1.72 (0.94-3.17) | 1.20 (0.29-5.02) |
| | Model 4 | 1.63 (0.25-10.51) | Ref | 1.51 (0.14-16.71) | 0.82 (0.38-1.76) | Ref | 0.51 (0.07-3.81) | 1.60 (0.90-2.86) | Ref | 1.55 (0.85-2.83) | 1.11 (0.27-4.61) |
| | Model 5 | 1.89 (0.30-11.84) | Ref | 1.50 (0.14-16.43) | 0.83 (0.39-1.76) | Ref | 0.52 (0.07-3.84) | 1.59 (0.89-2.83) | Ref | 1.53 (0.84-2.78) | 1.09 (0.26-4.56) |
| | Model 6 | 2.23 (0.31-15.88) | Ref | 1.71 (0.15-19.24) | 0.89 (0.42-1.89) | Ref | 0.53 (0.07-4.02) | 1.63 (0.92-2.90) | Ref | 1.44 (0.79-2.62) | 1.03 (0.25-4.30) |
| PSI | Model 3 | 1.96 (0.55-7.03) | Ref | - | 0.83 (0.42-1.64) | Ref | 1.50 (0.33-6.90) | 1.17 (0.64-2.14) | Ref | 1.88 (1.11-3.18) | 1.28 (0.39-4.17) |
| | Model 4 | 2.25 (0.55-9.20) | Ref | - | 0.88 (0.44-1.74) | Ref | 1.52 (0.35-6.73) | 1.18 (0.65-2.16) | Ref | 1.73 (1.03-2.88) | 1.21 (0.37-3.93) |
| | Model 5 | 1.93 (0.51-7.36) | Ref | - | 0.84 (0.43-1.64) | Ref | 1.69 (0.38-7.46) | 1.19 (0.65-2.18) | Ref | 1.70 (1.02-2.83) | 1.20 (0.37-3.87) |
| | Model 6 | 1.95 (0.55-6.92) | Ref | - | 0.79 (0.40-1.59) | Ref | 1.44 (0.32-6.42) | 1.21 (0.66-2.22) | Ref | 1.68 (1.00-2.81) | 1.15 (0.35-3.76) |
| FSIQ | Model 3 | 1.56 (0.23-10.58) | Ref | 1.35 (0.11-16.94) | 0.57 (0.19-1.67) | Ref | 3.02 (0.63-14.58) | 2.49 (1.32-4.70) | Ref | 1.63 (0.78-3.44) | 3.15 (1.05-9.44) |
| | Model 4 | 1.71 (0.20-14.90) | Ref | 1.28 (0.08-19.78) | 0.59 (0.20-1.75) | Ref | 2.31 (0.52-10.31) | 2.53 (1.34-4.78) | Ref | 1.39 (0.67-2.88) | 2.80 (0.95-8.24) |
| | Model 5 | 1.09 (0.15-7.72) | Ref | 2.11 (0.15-30.74) | 0.62 (0.21-1.83) | Ref | 2.31 (0.51-10.41) | 2.50 (1.33-4.72) | Ref | 1.40 (0.68-2.91) | 2.90 (0.99-8.51) |
| | Model 6 | 2.10 (0.28-15.95) | Ref | 2.44 (0.14-41.28) | 0.48 (0.14-1.63) | Ref | 2.81 (0.60-13.13) | 2.34 (1.22-4.50) | Ref | 1.43 (0.69-2.95) | 2.92 (0.99-8.64) |

Abbreviations: GWG: gestational weight gain, VCI: verbal comprehension index, VSI: visual space index, FRI: fluid reasoning index, WMI: working memory index, PSI: processing speed index, FSIQ: full scale intelligence quotient.

The cognitive level of average or above IQ was used as the reference group.

“-”: no results due to the limited cases. “Ref”: reference.

Model 3：Further adjusted for maternal pregnancy complications; Model 4：Further adjusted for birth weight Z scores by gestational age; Model 5：Further adjusted for children’s sex; Model 6：Further adjusted for exclusive breastfeeding for 6 months and the main caregivers before 3 years of age.
